# Ecological Drivers of Plasmid-Mediated Antimicrobial Resistance in Aquaculture

**DOI:** 10.1007/s00248-025-02684-0

**Published:** 2025-12-17

**Authors:** Laura E. Cota Ortega, Eduardo Quiroz-Guzmán, José Luis Balcázar

**Affiliations:** 1 Centro de Investigaciones Biológicas del Noroeste S.C, Av. Instituto Politécnico Nacional 195. Col. Playa Palo de Santa Rita Sur, La Paz, Baja California Sur 23096 Mexico; 2 Catalan Institute for Water Research (ICRA-CERCA), Girona, 17003 Spain

**Keywords:** Antimicrobial resistance, Aquaculture, Horizontal gene transfer, Microbial communities, Vibrio, Ecological resilience

## Abstract

Antimicrobial resistance (AMR) is a growing global challenge that compromises the effectiveness of disease control and increases risks for both human and animal health. Aquaculture systems are particularly vulnerable, as extensive and often inappropriate antimicrobial use has driven the emergence and persistence of multidrug-resistant bacteria. This mini-review summarizes the ecological and genetic mechanisms underlying AMR in aquaculture, with emphasis on plasmid-mediated resistance and its role in horizontal gene transfer. It also addresses the broader environmental and public health implications of these processes and calls for sustainable management, enhanced surveillance, and coordinated international policies to curb resistance dissemination and safeguard global food security.

## Implications of Antimicrobial Resistance

In recent years, the intensification and diversification of aquaculture have been constrained by the emergence and reemergence of diseases, frequently linked to unfavorable environmental conditions and inefficient management practices, including inadequate feeding, restocking, and nutrition [1]. These factors can lead to secondary bacterial infections, making the use of antimicrobial agents necessary for the treatment and prevention of infectious diseases in aquaculture. Among these, antibiotics are commonly applied as therapeutic, prophylactic, or metaphylactic agents [2, 3]. The most widely used antibiotics in aquaculture include amphenicols (florfenicol), β-lactams (amoxicillin), diaminopyrimidines (ormetoprim), macrolides (erythromycin), quinolones (oxolinic acid, flumequine, sarafloxacin, and enrofloxacin), sulfonamides (sulfadimethoxine), and tetracyclines (oxytetracycline) [1]. Although each country has its own regulations regarding the approval, usage, and residue limits of antibiotics in aquaculture products, the overuse and misuse of these compounds have driven the emergence of resistant bacteria [4].

**Fig. 1:**
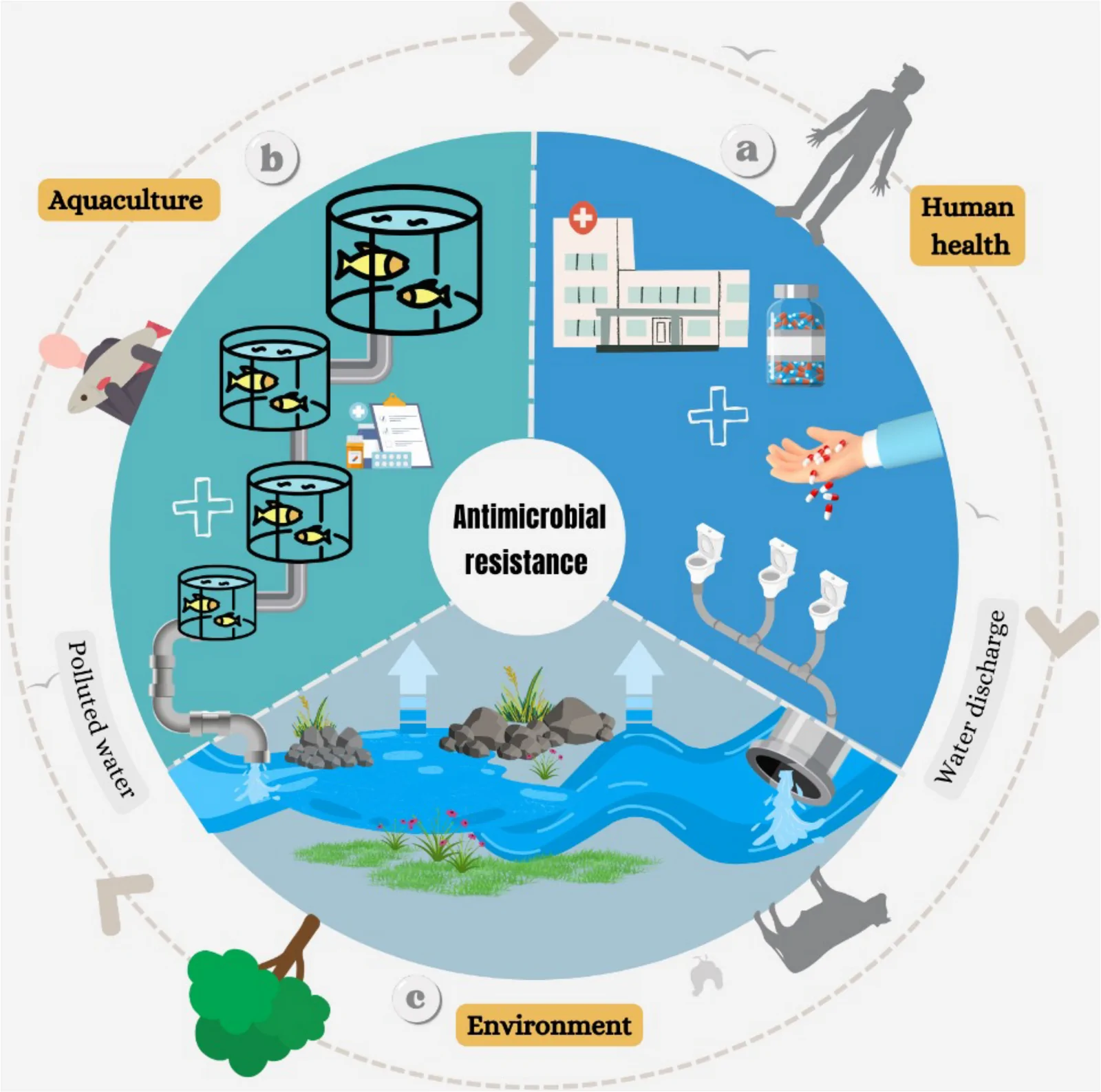
Conceptual model illustrating the interconnected pathways driving the emergence and dissemination of AMR across human, aquaculture, and environmental systems. (**a**) In human health, antibiotic use and wastewater discharge release resistant bacteria and antimicrobial residues into aquatic environments. (**b**) In aquaculture, antibiotic application for disease prevention and treatment promotes the selection of resistant strains and mobile genetic elements that can spread through water exchange. (**c**) The environment acts as a reservoir and transmission route, facilitating the circulation of resistance genes among microbial communities. Together, these interconnected processes create an ecological feedback system that sustains the selection, exchange, and environmental persistence of AMR determinants

Bacteria acquire resistance through genetic mutations or horizontal gene transfer, which allows them to share resistance traits even across species boundaries. Genetic flow dynamics influence the composition and adaptive potential of aquatic microbiomes, as mobile genetic elements such as plasmids, transposons, or bacteriophages play a key role in the evolution and ecology of bacterial communities by enabling both intra- and interspecies exchange of genetic information [5, 6]. For instance, a bacterium carrying a plasmid with antibiotic resistance genes (ARGs) can transfer it to another, conferring the same resistance [7, 8]. These ARGs can spread among diverse bacterial species, even among taxa not directly exposed to antibiotics. Under favorable conditions, resistant bacteria can emerge even in environments with limited antibiotic use, owing to their remarkable adaptability and capacity for gene exchange. The frequency of ARGs within bacterial populations increases rapidly when carriers gain a competitive advantage under antibiotic pressure [9]. Consequently, antimicrobial resistance (AMR) in aquaculture results from a combination of factors, including inappropriate antibiotic use, inadequate dosing, insufficient regulation, and limited disease management alternatives. Global pathogen dispersal and economic pressures to maximize production further intensify this problem. At the ecosystem level, AMR can be viewed as an emergent property of microbial networks responding to anthropogenic stressors. Figure 1 conceptualizes aquaculture as a key ecological driver of AMR, linking human antibiotic use, environmental reservoirs, and horizontal gene transfer dynamics within a single feedback-driven network that sustains resistance persistence and dissemination.

AMR in aquaculture has profound implications for human health and aquatic ecosystems [10].

The presence of antibiotic-resistant bacteria in aquaculture products can pose serious risks to consumers, as resistant strains may be transmitted through the food chain and compromise the effectiveness of antibiotic treatments [10, 11]. Moreover, antibiotic residues released into aquatic systems can trigger cascading ecological effects by disrupting the composition and function of microbial communities in water and sediment, thereby altering key biological processes. These disturbances may affect nutrient cycling, organic matter degradation, and microbial symbioses that are essential to ecosystem balance. Chronic exposure to antibiotics further imposes selective pressure on bacterial populations, enabling the persistence and spread of resistant strains [12]. AMR also increases the risk of cross-resistance, whereby ARGs are transferred among aquatic environments, the food chain, and human populations, highlighting a critical link between environmental integrity and human health. These multiple pathways emphasize the urgent need for integrated and sustainable approaches to mitigate AMR.

## Ecological Mechanisms of AMR: Plasmids as Key Drivers

Even at low concentrations, antibiotics can significantly disrupt microbial diversity in aquatic ecosystems. These disruptions may lead to ecological imbalances, degraded water quality, and long-term adverse effects on overall ecosystem health [13]. Consequently, their use must be carefully regulated to minimize the selection and spread of resistance, ensuring that treatments remain effective, sustainable, and environmentally safe, thereby protecting both public health and ecosystem integrity [14]. However, the prolonged and often excessive application of antibiotics continues to accelerate the emergence of AMR, constituting an increasing global concern.

Antibiotic resistance arises when microorganisms acquire the capacity to survive exposure to antimicrobial agents [15]. This process occurs through several key mechanisms, including genetic mutations, horizontal gene transfer, and biofilm formation [16–18] all of which compromise treatment efficacy and pose serious risks to environmental integrity, food safety, and human health. One major mechanism involves the modification of antibiotic targets, whereby bacteria alter essential cellular sites normally recognized by antibiotics, preventing drug binding and therapeutic action [19]. Reduced cellular permeability also contributes to resistance by limiting antibiotic uptake into bacterial cells. In addition, efflux pump systems enable the active removal of antimicrobial compounds before they reach effective intracellular concentrations. Another important mechanism is enzymatic degradation, in which bacteria produce enzymes capable of breaking down or inactivating antibiotics, thereby neutralizing their antimicrobial effects [20, 21].

Collectively, these processes not only enable bacteria to evade antibiotic action but also facilitate the spread of resistance through horizontal gene transfer mediated by mobile genetic elements [22, 23]. Although gene transfer can occur via transformation or transduction, plasmid-mediated conjugation is considered the most efficient process for the exchange of genetic material among bacteria. Conjugative plasmids act as ecological vectors of adaptation, linking genetic evolution with environmental resilience. They commonly harbor genes conferring adaptive traits, including metal tolerance, virulence factors, and AMR [24]. Understanding these plasmid-mediated processes is essential to interpreting the impact of environmental pressures on microbial networks and resistance evolution in aquaculture ecosystems.

Letchumanan et al. [25] conducted a comparative study on ARGs and plasmid profiles in shrimp and shellfish from Selangor, Malaysia, highlighting the consequences of extensive antibiotic use in aquaculture. They evaluated 385 *Vibrio parahaemolyticus* isolates for susceptibility to 14 antibiotics, reporting high resistance to ampicillin (85%), amikacin (66.8%), and kanamycin (50.1%), as well as to third-generation cephalosporins including cefotaxime (55.8%) and ceftazidime (34%). Plasmids were detected in 338 isolates, ranging from 1 to 7 per isolate and grouped into 27 distinct patterns. Similarly, Wang et al. [26] performed complete genome sequencing of *V. parahaemolyticus* strain Vp2015094, identifying two transmissible plasmids. One of them, plasmid pVp94-1, harboring multiple ARGs, conferred resistance to aminoglycosides (*aph(3’’)-Ib* and *aph(6)-Id*), β-lactams (*bla*_CARB−19_), diaminopyrimidines (*dfrA6*), fluoroquinolones (*qnrVC6*), phenicols (*floR*), sulfonamides (*sul2*), and tetracyclines (*tetB*,* tetM*,* tetR*, and *tetC*), and was flanked by transposase sequences that promote gene mobility. Furthermore, Xu et al. [27] investigated plasmid-mediated quinolone resistance in *Vibrio* spp., focusing on the *qnrS* gene, which encodes a protein that protects DNA gyrase and topoisomerase IV from quinolone inhibition. Among 1,811 foodborne *Vibrio* isolates, 34 (1.88%) carried the *qnrS* gene, predominantly the *qnrS2* allele. Strains harboring *qnrS* exhibited multidrug resistance, and conjugative pAQU-type plasmids carrying *qnrS2* facilitated resistance to ciprofloxacin and cephalosporins, indicating that the spread of these plasmids may accelerate the emergence of multidrug-resistant pathogens. These findings emphasize the importance of monitoring plasmids within integrated AMR surveillance programs to inform targeted prevention and control strategies across aquaculture systems.

Genomic evidence indicates that aquaculture environments not only accumulate resistance determinants but also facilitate the assembly of complex ARG platforms. Conjugative plasmids and integrative conjugative elements identified in *Vibrio* species frequently harbor multidrug resistance cassettes [22] and may include class 1 integrons associated with transposases, facilitating the recruitment and reshuffling of ARG clusters [28]. Enhanced plasmid heterogeneity and recombination patterns observed in aquaculture-associated bacterial populations relative to terrestrial production isolates suggest locally driven genetic assembly rather than simple acquisition from clinical or agricultural sources [29–32]. Consequently, aquaculture may act as an evolutionary hotspot in which novel ARG combinations can emerge prior to dissemination across environmental, food-chain, and clinical microbial networks [16, 33]. These ecological features of aquaculture systems help explain why these environments function as hotspots for AMR evolution.

## What Makes Aquaculture a Unique Hotspot for AMR Evolution

Aquaculture systems differ fundamentally from terrestrial livestock production systems in their potential to drive AMR emergence and dissemination. Unlike confined land-based farms, aquaculture operates as an open-water system in which treated effluents, uneaten medicated feed, fecal material, and associated microbial communities are continuously exchanged with surrounding aquatic environments. This open connectivity facilitates the dispersal of antimicrobial residues, resistant bacteria, and mobile genetic elements beyond production boundaries and into connected aquatic ecosystems [16, 33].

High stock densities, chronic physiological stress, organic matter enrichment, and frequent prophylactic antibiotic use impose strong and sustained selective pressure for resistance development. These stressors intersect with highly complex aquatic microbial assemblages composed of environmental bacteria, commensal microbiota, and opportunistic pathogens, thereby promoting intense cross-species interactions and genetic exchange. Such multispecies contact zones are especially conducive to horizontal gene transfer, particularly through plasmid-mediated conjugation.

Biofilms forming on tank surfaces, sediments, piping systems, and particulate organic matter function as “genetic hotspots”, concentrating bacterial cells at high densities and enhancing plasmid exchange efficiency [34]. In addition, the presence of heavy metals such as copper and zinc, commonly applied as antifouling biocides or dietary supplements in aquaculture, generates co-selection pressures that stabilize resistance plasmids carrying both metal- and antibiotic-resistance genes [22, 33].

Compared to terrestrial livestock systems, aquaculture therefore represents a uniquely efficient ecological amplifier for AMR development, combining open-system dispersal, intense selective pressure, dense multispecies microbial contact networks, and biofilm-driven genetic exchange. These integrated ecological drivers define aquaculture not as static, but as an active reservoir for the assembly of mobile genetic elements carrying ARGs with high potential for global dissemination.

## Antibiotic-Resistant Pathogens in Aquaculture: ***Vibrio*** Species as Key Case Studies

AMR has been documented in a broad range of aquaculture-associated pathogens. *Aeromonas hydrophila* isolates from tilapia, carp and other cultured fish frequently display resistance to quinolones, tetracyclines and β-lactams, with mobile genetic elements, including plasmids, contributing to resistance dissemination [35]. Likewise, multidrug-resistant *Flavobacterium columnare*, the etiological agent of columnaris disease, has been widely reported in freshwater hatcheries, showing variable resistance profiles and evidence of horizontally acquired resistance determinants [36]. In addition, multidrug-resistant *Edwardsiella tarda* isolates from aquaculture environments have been documented, including transferable plasmids carrying aminoglycoside and tetracycline resistance genes [37, 38]. These examples demonstrate that AMR is pervasive among bacterial fish pathogens, although the prevalence, mobility, and genomic organization of resistance determinants can vary substantially among taxa.

Despite the importance of these pathogens, *Vibrio* species are particularly relevant opportunistic organisms for investigating the contribution of plasmid-mediated mechanisms to AMR in aquaculture. Members of this genus are ubiquitous in marine settings, frequently dominate microbial communities within production systems, and infect a wide range of fish and shellfish species [39]. In addition, several *Vibrio* lineages include important zoonotic strains affecting human health [40, 41]. Their broad host range, environmental persistence, and frequent association with transferable resistance determinants make *Vibrio* a key genus for understanding how AMR emerges and spreads across the One Health interface.

An early study on plasmid tracking in *Vibrio* species was conducted by Molina-Aja et al. [29], who investigated plasmid occurrence and its relationship to AMR in strains isolated from diseased shrimp. Their results showed that 80% of strains carried plasmids, grouped into 11 distinct profiles, with 70% exhibiting resistance to carbenicillin and ampicillin.

In recent decades, advances in genomics have refined AMR research, allowing more precise characterization of resistance genes and their transmission mechanisms. Prabina et al. [42] studied the prevalence of antibiotic-resistant *Vibrio* species in diseased Pacific white shrimp (*Penaeus vannamei*) from Ernakulam, India. They analyzed *V. alginolyticus*, *V. cholerae*, *V. fluvialis*, *V. mimicus*, and *V. parahaemolyticus*, together with *Aeromonas* spp. and *Shewanella alga*e. The study identified *V. alginolyticus* as the most resistant isolate, with a multiple antibiotic resistance (MAR) index of 0.60, followed by *V. mimicus* (0.54) and *V. parahaemolyticus* (0.42). The MAR index measures the proportion of antibiotics to which a bacterium is resistant, with higher values indicating greater resistance levels. Haque et al. [43] reported similar findings in Bangladesh, where *Vibrio* isolates from shrimp and surrounding environments exhibited resistance rates ranging from high (92.2%) to moderate (15.7%) for ampicillin, amikacin, cefotaxime, tetracycline, ceftazidime, gentamicin, nalidixic acid, levofloxacin, and ciprofloxacin, and low resistance (3.9%) to imipenem, meropenem, chloramphenicol, and trimethoprim-sulfamethoxazole. Notably, 52.9% of isolates were multidrug-resistant, with a MAR index of 1.0. Similarly, Yu et al. [44] assessed the population distribution and antibiotic resistance of five pathogenic *Vibrio* species in Pacific white shrimp breeding systems in China. They obtained 418 isolates, 312 of which belonged to the genus *Vibrio*, with *V. alginolyticus*,* V. harveyi*,* V. parahaemolyticus*,* V. cholerae*, and *V. campbellii* as the dominant species. Among the resistant isolates, four ARGs were detected: *strA* (43.8%), *strB* (11.7%), *sul2* (8.8%), and *qnrVC* (2.9%).

## Tackling the AMR Crisis

Effectively addressing AMR in aquaculture requires a multilevel approach that combines robust regulatory policies, responsible antimicrobial stewardship, and coordinated action among scientific institutions, governmental bodies, and industry stakeholders [45–47]. This integration should foster ecosystem-based management frameworks capable of reconciling aquaculture productivity with ecological sustainability and public health protection. The incorporation of microbial ecological principles into these frameworks can enhance our ability to anticipate resistance emergence, identify key environmental risk factors, and develop adaptive control strategies. Likewise, harmonizing regulatory standards across local, national, and international levels remains essential to ensure the consistent implementation of antibiotic control policies and compliance throughout global supply chains.

At the production level, priority should be given to management practices that minimize disease risk while avoiding reliance on routine chemotherapeutic interventions. Optimizing stocking densities, improving water quality, and stabilizing rearing conditions can substantially reduce physiological stress in cultured organisms, thereby decreasing susceptibility to infection. In addition to environmental control, strategies focused on restoring or maintaining beneficial microbial balance through habitat management or the application of microbial consortia may further enhance resistance to pathogen colonization. Preventive measures such as vaccination programs, probiotic supplementation, and strengthened biosecurity protocols further diversify disease control options while reducing selective pressures acting on bacterial populations. Complementary approaches, including immune-enhancing diets, selective breeding for disease resistance, and habitat enrichment, can additionally strengthen system resilience against infectious outbreaks. Notably, several intervention strategies have already demonstrated measurable reductions in antibiotic dependency within commercial aquaculture. Vaccines targeting *Aeromonas* spp., *Lactococcus garvieae*, and *Vibrio* spp. have been associated with substantial decreases in antimicrobial treatments across multiple fish farming systems [48–50]. Similarly, probiotic applications in shrimp culture systems have effectively limited the incidence of *Vibrio* infections while contributing to lower antibiotic input levels [51, 52]. Experimental trials investigating bacteriophage therapy against pathogenic *Vibrio* spp. and *Aeromonas* spp. have reported promising prophylactic and therapeutic outcomes under aquaculture conditions, highlighting the potential of targeted biological control as an alternative to conventional antimicrobial use [53, 54].

Equally critical is the responsible and well-regulated use of antibiotics, guided by precise dosing schemes and clearly defined treatment durations. Routine monitoring programs should ensure that antimicrobial application remains targeted and evidence-based, thereby minimizing unnecessary exposure to broad-spectrum compounds. Investment in advanced diagnostic and molecular tools, including PCR-based pathogen detection, metagenomic surveillance, and rapid biosensor technologies, would facilitate early disease identification and enable timely intervention to limit resistance development.

Capacity-building initiatives for producers are also fundamental, as informed decision-making strongly influences antibiotic stewardship. Farmers should understand not only the technical principles of disease management but also the broader ecological and economic consequences of antimicrobial misuse. In this context, institutional measures including mandatory veterinary prescriptions, traceability systems for antibiotic distribution, and periodic audits of farm practices are needed to ensure transparency and accountability. Financial incentives and certification schemes that promote sustainable management may further encourage compliance with responsible production standards.

AMR in aquaculture remains a complex, evolving challenge that requires sustained interdisciplinary collaboration among researchers, policymakers, and producers. Future research should therefore prioritize the integration of ecological, genomic, and epidemiological datasets to refine predictive risk models and evaluate the long-term effectiveness, scalability, and feasibility of existing mitigation strategies. In addition, systematic assessments of the ecological safety and cost-effectiveness of non-antibiotic disease control tools across diverse production systems remain key research priorities.

Strengthening public awareness of antimicrobial misuse and fostering transparent communication among producers, scientists, consumers, and regulatory bodies remains essential to support socially driven transitions toward sustainability. Through this integrated, preventive, and evidence-based framework, aquaculture can continue to develop in ways that protect food security while safeguarding the ecological integrity of aquatic ecosystems.

Taken together, these principles can be translated into the following priority actions to support the mitigation of AMR across aquaculture systems:

- Implementation of targeted vaccination programs to reduce the incidence of bacterial disease and the subsequent need for antibiotic treatments.
- Application of probiotic strategies and microbiome management approaches to enhance microbial stability and suppress pathogen proliferation.
- Improvement of husbandry and environmental management practices to lower disease pressure through optimized stocking densities, water quality, and rearing conditions.
- Molecular surveillance of ARGs and mobile genetic elements, including plasmids, to enable early detection of emerging resistance trends.
- Strengthened regulatory oversight of antimicrobial use and residue monitoring to ensure compliance with stewardship policies along the production chain.
- Education and training programs for producers and veterinarians to promote evidence-based disease management and responsible antimicrobial practices.

## Perspectives

The genetic diversity observed among resistant strains, together with their geographic distribution, highlights the importance of bacterial mobility and cross-border exchange in driving the evolution of AMR. The detection of ARGs in aquaculture-associated bacteria raises significant biosecurity and public health concerns, emphasizing the urgent need for enhanced genomic surveillance, risk assessment, and preventive management strategies. From a microbial ecology perspective, these observations further reveal the interconnectedness of microbial communities across aquatic and terrestrial systems, linking local selection events to global resistance networks.

Expanding our understanding of these mechanisms requires an integrated approach that combines molecular epidemiology, ecological modeling, and data-driven monitoring to trace the origins and dissemination routes of resistance determinants. Such multidisciplinary frameworks will help clarify how ecological interactions, including competition, cooperation, and horizontal gene flow, govern resistance evolution at the ecosystem scale. Strengthened cooperation among scientists, policymakers, and industry stakeholders will be essential for translating research findings into coordinated biosecurity measures and evidence-based regulations.

In the long term, advancing sustainable practices in aquaculture through innovation, education, and global collaboration will play a pivotal role in reducing antimicrobial dependence, safeguarding aquatic environments, and strengthening both food security and public health resilience.

## Data Availability

No datasets were generated or analysed during the current study.
